# In Situ Visualization of Electron Beam‐Driven High‐Entropy Alloy Crystallization

**DOI:** 10.1002/advs.202512587

**Published:** 2025-10-21

**Authors:** Azadeh Amiri, Reza Shahbazian‐Yassar

**Affiliations:** ^1^ Department of Mechanical and Industrial Engineering University of Illinois Chicago Chicago IL 60607 USA

**Keywords:** high entropy alloy nanoparticles, in situ transmission electron microscopy

## Abstract

Achieving compositionally uniform high‐entropy alloy (HEA) nanoparticles via reduction‐based synthesis remains challenging due to variations in elemental reduction, diffusion, and phase stability. Using in situ transmission electron microscopy (TEM), this study visualizes the electron beam–induced crystallization of amorphous high‐entropy glycerolate (HE‐glycerolate) films composed of Mg, Mn, Co, Ni, and Zn. The transformation proceeds through phase separation, radiolytic reduction, and localized atomic rearrangement, producing single‐phase face‐centered cubic (fcc) HEA nanoparticles with uniform cuboidal morphology and dominant {100} facets. Compared to thermal annealing, the electron beam pathway offers finer control over composition and morphology by limiting atomic mobility and preventing phase segregation or Co/Ni clustering. This displacement‐driven, athermal process enables gradual, diffusion‐limited crystallization within confined regions, resulting in well‐defined, compositionally homogeneous alloys. The study reveals the mechanism of electron beam‐driven crystallization of HEA nanoparticles and establishes a broader principle that controlling atomic mobility is key to achieving stable, multielement solid solutions. The insights gained, highlighting the role of confined atomic mobility, offer a valuable foundation for designing new low‐temperature synthesis routes for uniform HEA materials with controlled phase and morphology, and inform the development of scalable processing strategies for homogeneous multicomponent systems.

## Graphical abstract

In situ transmission electron microscopy visualizes the electron beam‐driven crystallization of amorphous multielement glycerolate film into single‐phase high‐entropy alloy (HEA) nanoparticles. The athermal process proceeds via radiolytic reduction and localized atomic rearrangement, forming compositionally uniform, cuboidal face‐centered cubic HEA nanostructures while suppressing elemental segregation through diffusion‐limited crystallization.

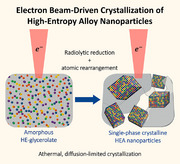

## 1 Introduction

High entropy alloys (HEA) represent a fascinating frontier in material science, characterized by a disordered single‐phase crystal structure composed of five or more elements.^[^ ^1^, ^2^, ^3^, ^4^ ^]^ These alloys offer a unique combination of properties, including high strength, excellent corrosion resistance, and enhanced mechanical properties, making them promising candidates for various applications.^[^ ^5^, ^6^, ^7^ ^]^ However, the synthesis of HEAs presents significant challenges, particularly when attempting to incorporate multiple elements with diverse properties into a single‐phase solid solution alloy and prevent chemical phase segregation.^[^ ^8^, ^9^ ^]^ Wet chemistry methods offer a straightforward and potentially scalable approach to producing high‐yield metal and alloy at a low cost; however, they are notably sensitive to various experimental conditions, including precursor selection, solvent systems, surfactants, and temperature.^[^ ^10^, ^11^, ^12^, ^13^ ^]^ The simultaneous reduction and integration of metals with different ion complexation behaviors, reduction potentials, and reaction kinetics adds further complexity.^[^ ^14^, ^15^, ^16^ ^]^ Achieving a homogeneous, single‐phase solid solution requires careful precursor design to control composition and prevent phase segregation.^[^ ^14^, ^17^, ^18^, ^19^, ^20^ ^]^ Among various strategies, glycerol‐based sol‐gel synthesis has emerged as a promising approach. Glycerol effectively coordinates with multiple metal ions via its hydroxyl groups, forming high‐entropy glycerolates (HE‐glycerolates).^[^ ^21^, ^22^, ^23^, ^24^, ^25^, ^26^, ^27^ ^]^ These amorphous intermediates enable uniform metal ion distribution and have been widely used to produce high‐entropy oxides, sulfides, hydroxides, and phosphides through thermal treatment.^[^ ^28^, ^29^, ^30^, ^31^, ^32^, ^33^ ^]^ When subjected to reductive conditions, the same precursors have the potential to yield metallic HEAs, suggesting a possible route for alloy synthesis that warrants further investigation.

Building upon the potential of HE‐glycerolate precursors for metallic alloy formation, understanding the transformation of these amorphous materials into crystalline HEA phases is critical.^[^ ^34^, ^35^, ^36^, ^37^ ^]^ While thermal treatment is traditionally used for such transformations, electron beam irradiation within a transmission electron microscope (TEM) offers a powerful alternative.^[^ ^38^ ^]^ This technique induces crystallization and allows direct, real‐time tracking of microstructural changes, offering valuable insight into nanoparticle development.^[^ ^39^, ^40^ ^]^ Electron beam‐induced synthesis is distinct from conventional methods such as electron beam deposition or thermally assisted crystallization. Unlike thermal methods, which rely on bulk heating and diffusion, this athermal pathway involves localized energy transfer via different electron–specimen interactions, including knock‐on displacement and radiolytic effects, initiating structural transformation without a significant rise in temperature (less than 50 °C under typical TEM imaging conditions^[^ ^41^, ^42^ ^]^).^[^ ^38^, ^43^, ^44^, ^45^ ^]^ High‐energy electron irradiation initiates structural transformation through a combination of elastic and inelastic interactions.^[^ ^38^ ^]^ Atomic displacement occurs when the momentum transferred during elastic collisions exceeds the displacement threshold of atoms, an effect most pronounced for lighter elements and under‐coordinated surface atoms. In regions where bonding is weakest, this can lead not only to displacement but to complete ejection of atoms from the surface, known as sputtering. Both mechanisms contribute to the formation of defects, local compositional changes, and enhanced atomic mobility.^[^ ^46^, ^47^ ^]^ Inelastic interactions further modify the matrix through radiolytic effects, where energy absorption leads to ionization and bond disruption.^[^ ^48^ ^]^ These processes destabilize the amorphous network, promoting structural rearrangement and initiating crystallization. Specimen charging may alter local electrostatic fields, and the emission of secondary electrons amplifies radiolytic activity through cascades of low‐energy interactions.^[^ ^49^ ^]^ Together, these effects act synergistically to drive athermal and localized crystallization under electron beam exposure.

Electron beam‐induced crystallization has been studied across various amorphous substrates. In many studies, inorganic glassy amorphous thin films such as chalcogenide glasses (e.g., Ge_2_Sb_2_Te_5_) or transition metal‐based amorphous silicon carbides (e.g., Zr‐Si‐C) are used as precursor materials.^[^ ^50^, ^51^, ^52^, ^53^, ^54^ ^]^ In such amorphous systems with covalent or covalent–metallic bonding structure, knock‐on displacement effect of electron beam and atomic structural rearrangement are the primary drivers of the amorphous to crystalline phase transition.^[^ ^53^, ^54^, ^55^ ^]^ In contrast, metal‐organic precursors such as metal‐organic frameworks and coordination polymer glassy materials are hybrid systems in which metal ions are bonded through coordination with organic ligands.^[^ ^56^ ^]^ In these systems, the crystallization process is generally initiated by radiolytic decomposition of the matrix and followed by reduction of metal ions, which is a prerequisite for nucleation.^[^ ^57^, ^58^ ^]^ Studies on metal‐organic precursors have been relatively limited and mostly focused on single‐metal systems.^[^ ^57^, ^59^, ^60^, ^61^ ^]^ Nonetheless, they demonstrate the potential of these materials for synthesizing crystalline metals and alloys, while presenting opportunities to explore alternative mechanistic pathways, particularly where processes deviate from the conventional two‐step framework.

The formation pathways of crystalline HEA nanoparticles from an amorphous mixture of polymer‐metal salts under electron‐beam synthesis have not been reported before. The presence of several types of metal ions and organic molecules adds further complexity due to the interplay of multiple elements, requiring careful consideration of their individual and collective interactions during synthesis. Leveraging the promising capabilities of electron beam‐induced synthesis of nanoparticles, this study investigates the nucleation and growth of crystalline multielement nanoparticles from an amorphous HE‐glycerolate precursor using the in situ TEM technique.

## 2 Results and Discussion

**Figure 1:**
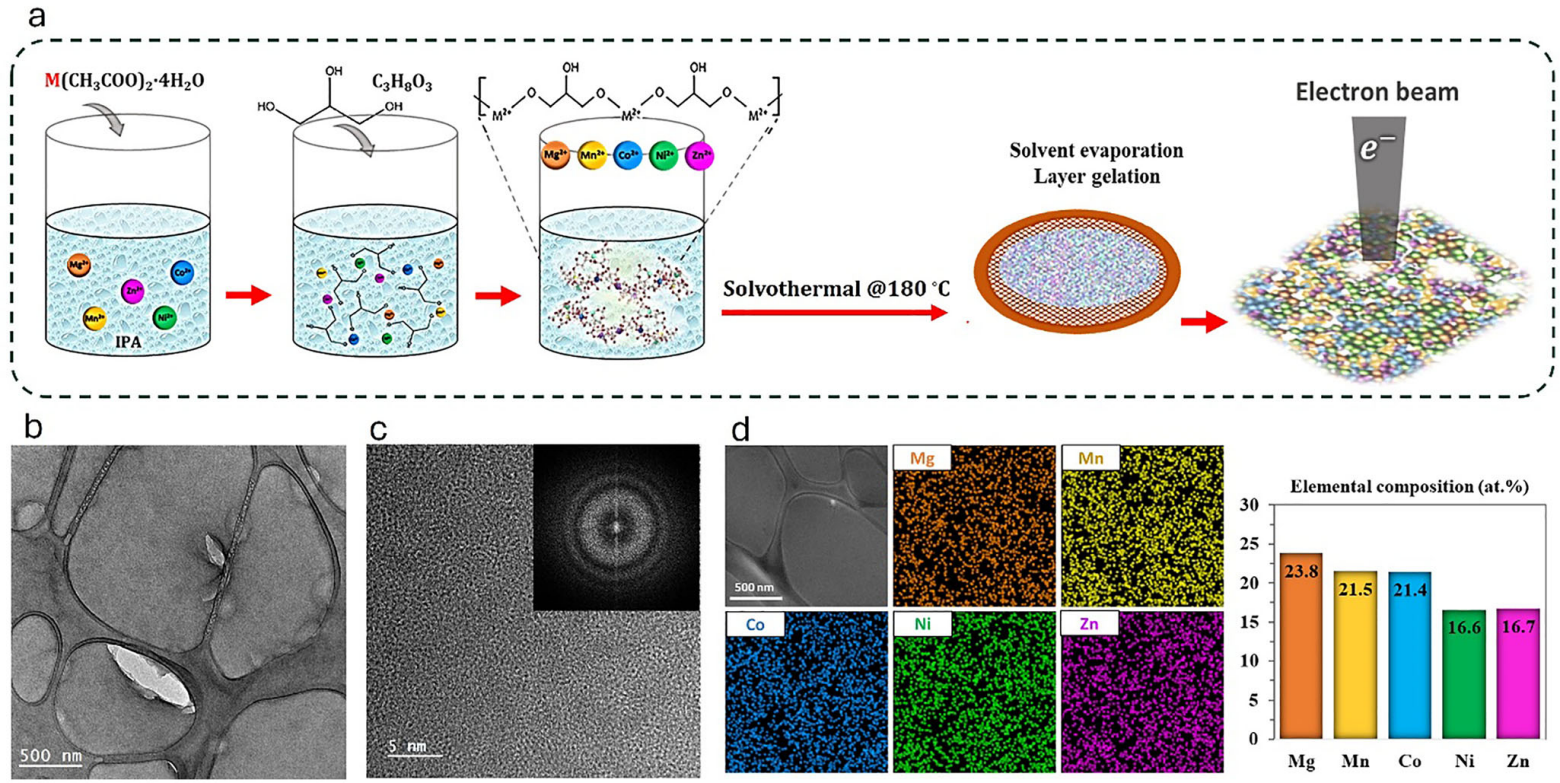
Preparation and characterization of HE‐glycerolate film sample. a) Schematic illustrating the experimental procedure for the preparation of the MgMnCoNiZn‐glycerolate solution and the subsequent thin film deposition on a TEM grid. b) TEM image of the deposited HE‐glycerolate thin film. c) HRTEM image and corresponding FFT diffractogram (inset) showing the initial amorphous phase structure of the film. d) STEM‐EDS compositional characterization of the deposited film, including elemental mapping and quantitative atomic percent composition.

Viscous solutions of HE‐glycerolate containing five metal elements (Mg, Mn, Co, Ni, Zn) coordinated with polymerized glycerol (see Experimental Procedure Section) were deposited onto a lacy carbon copper TEM grid and dried under ambient conditions to form thin films (**Figure** **1**a). The TEM image in Figure 1b reveals the as‐deposited HE‐glycerolate film to be amorphous and free‐standing between the lacy carbon supports. A thinner area within the film is observed, suggesting damage to the top layers after drying and evaporation of volatile liquids, including isopropyl alcohol (IPA) and water from the dehydration of the initial hydrated phase of the metal acetates during the solvothermal process. Excess glycerol, being non‐volatile, may remain in its liquid form in the film. The high‐resolution TEM (HRTEM) image at higher magnification, along with the corresponding Fast Fourier Transformed (FFT) diffractogram (Figure 1c), confirms the amorphous structure of the as‐deposited film. Further characterization by energy dispersive X‐ray spectroscopy (EDS) in scanning transmission electron microscopy (STEM) demonstrates a homogeneous and near‐equimolar distribution of the five metal cations (24.8 at.% Mg, 21.5 at.% Mn, 21.3 at.% Co, 15.6 at.% Ni, and 16.7 at.% Zn) within the HE‐glycerolate film (Figure 1d). The incorporation of multiple metal ions with varying coordination modes to glycerolate ligands is known to promote amorphous characteristics in the HE‐glycerolate structure.^[^ ^28^ ^]^ The HE‐glycerolate solution remains uniform and colloid‐free after 1 h of solvothermal processing, whereas extended processing times (>6 h) lead to the formation of amorphous glycerolate spheres (Figure S1, Supporting Information). The HE‐glycerolate solution also demonstrates long‐term stability, remaining stable and homogeneous for over a year at room temperature (Figure S2, Supporting Information), whereas single‐element solutions tend to form precipitates over time, varying by element. Additional characterizations of amorphous HE‐glycerolate are reported in our previous work.^[^ ^30^ ^]^

**Figure 2:**
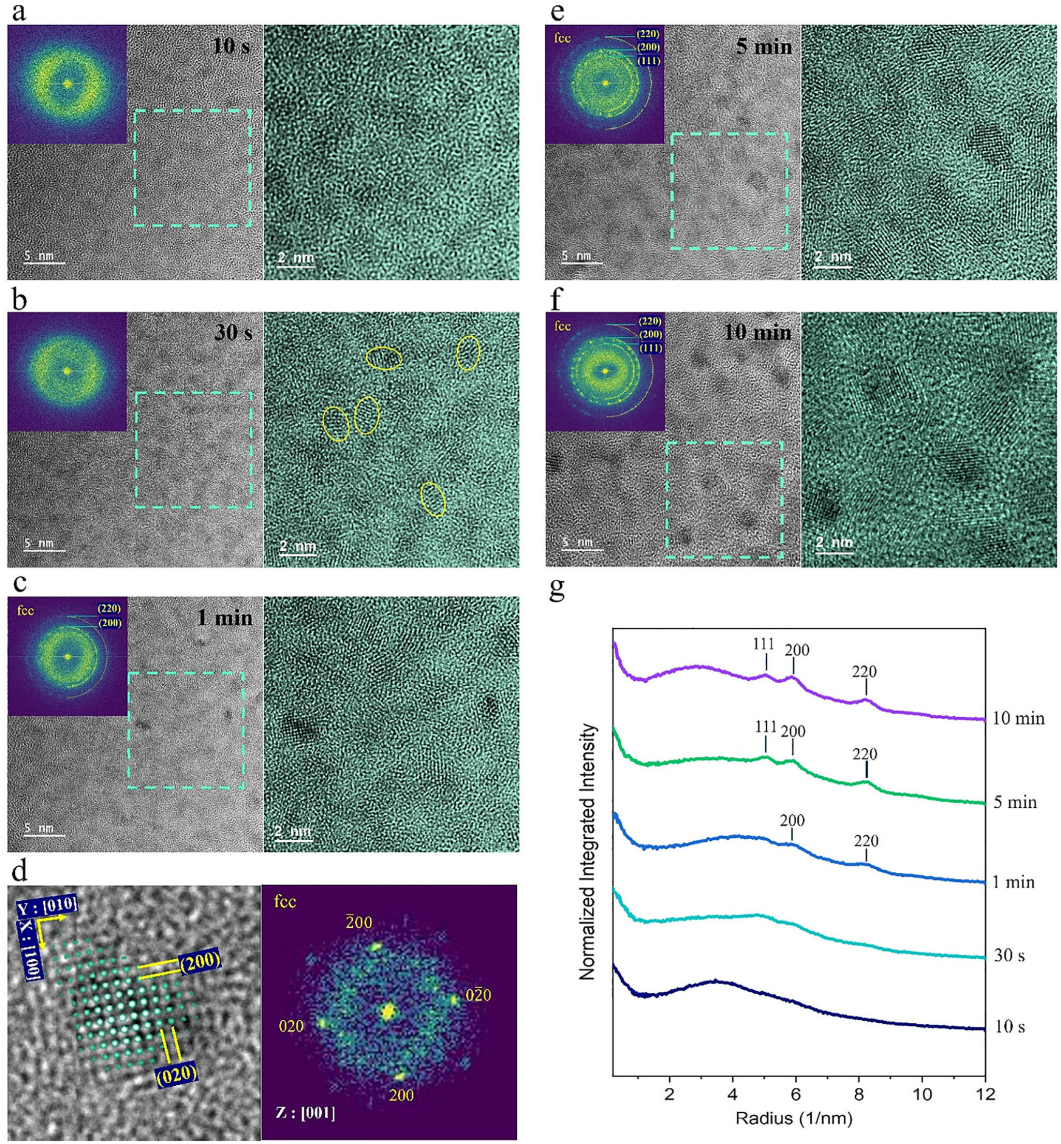
In situ TEM observation of crystallization process of multi‐element nanoparticles from the amorphous HE‐glycerolate film under electron beam irradiation. The HRTEM images, corresponding FFT diffractograms (inset), and false‐colored magnified images (right) of the areas marked by dashed squares, illustrate the microstructural evolution at different time points: a) 10 s, b) 30 s, c, d) 1 min, e) 5 min, and f) 10 min. (d) A magnified HRTEM region at t = 1 min, showing a small cuboidal crystalline domain, with the FFT pattern displaying an fcc structure along the [001] zone axis. g) Radial intensity profiles extracted from the FFT images show the amorphous profile at the initial stage and the emergence of diffraction peaks corresponding to the {111}, {002}, and {022} planes of the fcc structure over time.

The in situ crystallization of the as‐deposited HE‐glycerolate film in TEM, shown in **Figure** **2**, reveals a gradual structural transformation under continuous electron beam exposure (5 × 10^6^ e nm^−2^.s dose rate). Initially, the HE‐glycerolate film presents a uniform amorphous phase, identified by the broad diffuse halo ring in the FFT and uniform phase contrast across the film (t = 0 s, Figure 1C). Shortly after electron beam irradiation (t = 10 s, Figure 2a), phase separation occurs, indicated by contrast variation due to phase inhomogeneity within the amorphous matrix, resulting in two amorphous sub‐regions: metal‐rich (dark gray) and metal‐poor or organic‐rich (bright gray). This phase separation is likely driven by the redistribution of atomic species and local structural fluctuations under the influence of electron beam interactions. At t = 30 s (Figure 2b), the early stage of crystallization is detected, with short‐range ordering appearing within the metal‐rich regions, highlighted by circled areas in the figure. At t = 1 min (Figure 2c), the emergence of sub‐2 nm crystalline nanoparticles embedded within the amorphous matrix is observed. The FFT of the entire region displays a combination of diffuse amorphous rings and low‐intensity crystalline diffraction spots, reflecting the coexistence of amorphous and nanocrystalline phases. The corresponding crystalline diffraction spots in FFT reflect interplanar spacings of ≈1.7 and 1.2 Å, associated with {200} and {220} planes of face‐centered cubic (fcc) Ni and Co, suggesting the nucleation of Ni/Co‐like fcc clusters. The localized FFT analysis of a selected nanoparticle (Figure 2d) further supports the [100]‐oriented Ni/Co‐like fcc structure, indicated by the observed interplanar spacings and diffraction symmetry. These findings suggest that Ni and Co atoms may act as effective nucleation centers, templating the crystal lattice and directing atomic rearrangement. Prolonged electron irradiation further enhances crystallization. After 5 min of electron beam exposure, additional lattice fringes appear in the TEM image, with an increase in the average crystalline nanoparticle size to ≈3 nm (Figure 2e). The number and intensity of diffraction spots in the corresponding FFT also increase, indicating a more visible crystalline phase. The FFT pattern at this stage is consistent with an fcc structure, with three diffraction rings corresponding to the {111}, {002}, and {022} planes. By t = 10 min (Figure 2f), the crystallinity is further enhanced, with a more pronounced phase contrast between the crystalline domains and the surrounding amorphous matrix. The FFT shows sharp and well‐defined diffraction spots, confirming the continued evolution of the crystalline phase, as well as a remaining faint diffuse ring indicative of residual amorphous regions. The radial intensity profile plots derived from the FFT images (Figure 2g) also illustrate the initial amorphous nature of the film, followed by the emergence of distinct crystalline peaks corresponding to the fcc structure after 1 min of electron beam exposure. Even after 10 min, a broad, low‐intensity amorphous feature remains visible, indicating an incomplete transition to the full crystalline phase.

The observed electron beam‐induced crystallization follows a two‐step transformation mechanism, involving initial precursor decomposition followed by atomic rearrangement. The amorphous HE‐glycerolate film is composed of polymerized metal ion glycerolate nanosheets containing carbon, oxygen, hydrogen, and metal ions (Mg, Mn, Co, Ni, Zn).^[^ ^30^ ^]^ The interactions of the electron beam with the specimen are known to induce structural changes through mechanisms such as radiolysis and charge transfer, knock‐on displacement.^[^ ^38^ ^]^ Radiolytic interactions break down the substrate's chemical structure, creating localized defect sites that facilitate atomic rearrangement. Particularly, the radiolytic reduction of metal ions to zero‐valent atoms disrupts their ionic and covalent interactions with glycerolate ligands, contributing to matrix decomposition and promoting defect formation. Reduced single metal atoms exhibit relatively lower displacement energies compared to their bonded ionic states, enhancing their mobility and facilitating diffusion toward nucleation sites.^[^ ^46^ ^]^ The preferential nucleation of fcc clusters is likely attributed to the higher reduction potentials of Ni and Co and their primary reduction, which enable these metal atoms to accumulate at defect sites and initiate crystallization earlier than other metal species. Simultaneously, knock‐on atomic displacement plays a significant role in this process, occurring when the energy transferred from the electron (E_T_) exceeds the atomic displacement energy of the material (E_d_) to displace atoms within the specimen matrix. These interactions drive defect generation, vacancy formation, and eventual atomic reorganization into crystalline structures. The maximum transferable energy (E_T_) between the electron and the atom depends on both the atomic number and the electron acceleration voltage.^[^ ^38^, ^47^ ^]^ The displacement energy (E_d_) is material‐specific, generally lower for lighter elements and surface atoms compared to more stable bulk atoms. Thus, lighter elements and surface atoms are particularly more prone to displacement and even sputtering if the transferred energy approaches their sublimation energy.^[^ ^62^ ^]^ Based on the calculated maximum transferable energy (E_T_) from the incident 200 keV electron for each element and their estimated displacement energies (E_d_) provided in Table S1 (Supporting Information), it is evident that lighter elements like hydrogen, oxygen, and carbon receive significantly higher transferred energy compared to metal atoms.^[^ ^62^, ^63^ ^]^ The higher energy transferred to lighter organic elements makes them more susceptible to displacement and contributes to the formation of defects and local structural instabilities within the matrix. This preferential displacement of lighter elements induces density fluctuations and enhances atomic mobility, facilitating the phase separation and crystallization observed in the TEM images. The generated vacancies and under‐dense regions act as channels for metal atom diffusion, enabling the metal atoms to aggregate and contribute to the crystallization process. Secondary interactions, where displaced lighter atoms transfer energy to metal atoms, may further enhance metal atom mobility, promoting the nucleation and growth of crystalline phases.^[^ ^51^, ^64^ ^]^ Although the thermal effects from inelastically scattered electrons may not directly initiate crystallization, they likely accelerate atomic diffusion, contributing to a more rapid phase transformation.^[^ ^38^ ^]^ As irradiation continues, atomic displacement and radiolytic decomposition accelerate the amorphous‐to‐crystalline transition in the metal‐rich regions. The two‐step structural transformation also aligns with the histogram analysis of contrast distribution in Figure S3 (Supporting Information), showing a rapid broadening of the narrow single peak of a more uniform amorphous reflecting increased heterogeneity due to phase separation up to t = 30s. The rate of broadening slows thereafter but continues throughout the crystallization. This indicates that the process follows the two processes: initial phase separation followed by gradual progressive crystallization.

**Figure 3:**
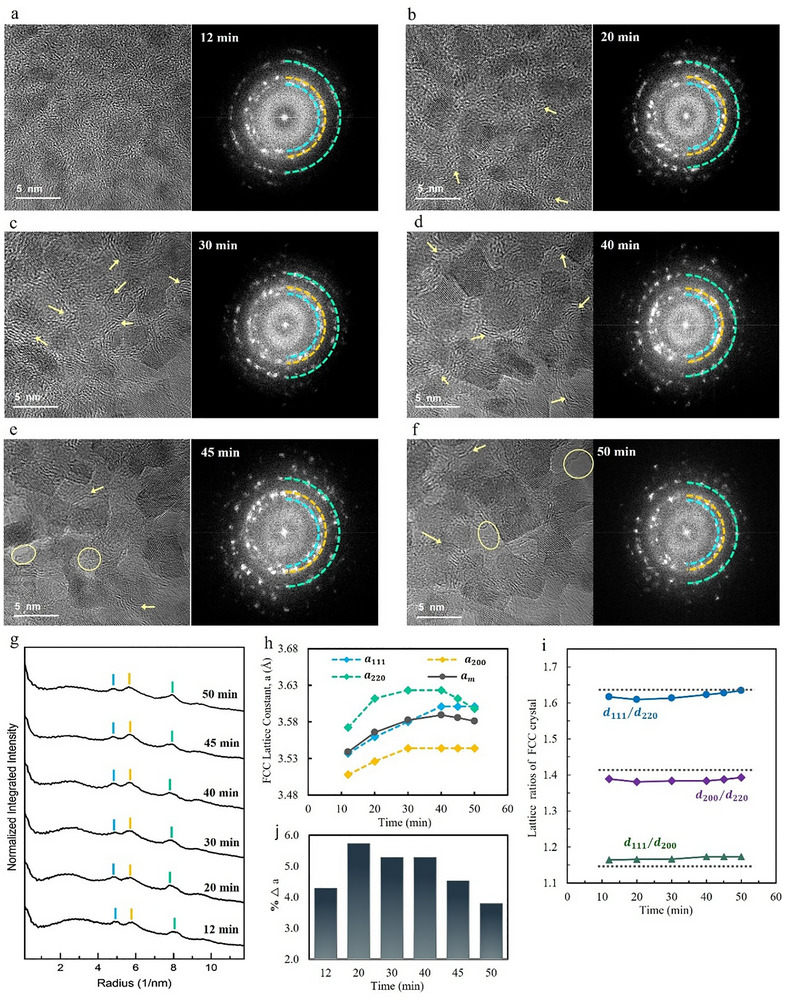
Microstructural evolution and lattice parameter variation during electron beam‐induced crystallization. HRTEM images (left) and corresponding FFT diffractograms (right) showing the progression of crystallization at different time points: a) 12 min, b) 20 min, c) 30 min, d) 40 min, e) 45 min, and f) 50 min. Yellow arrows highlight the curved striations associated with the short‐range alignment of the polymeric matrix, while yellow circles denote the development of pores within the amorphous matrix as it decomposes. g) Radial intensity profiles extracted from FFTs, illustrating the evolution of diffraction peaks corresponding to fcc lattice reflections. h) The calculated directional fcc lattice constants (a_111_, a_200_, a_220_) and their average (a_m_) over time. i) Evolution of lattice *d*‐spacing ratios of d_111_/d_200_, d_200_/d_220,_ and d_111_/d_220_ relative to ideal fcc values (dashed lines, d_111_/d_200_ = 1.155, d_200_/d_220_ = 1.414, and d_111_/d_220_ = 1.633), revealing anisotropic strain in the crystal structure. j) Percentage deviation in lattice constant (%Δa) over time.

The microstructural evolution of the HE‐glycerolate film and the resulting crystalline nanoparticles under prolonged electron beam irradiation is shown in **Figure** **3**. High‐resolution (4k × 4k) TEM imaging captured the gradual transformation of the film over 50 min, with TEM images and corresponding FFT diffractograms recorded at 12, 20, 30, 40, 45, and 50 min (Figure 3a–f). The radial intensity profile plots of the FFTs (Figure 3g) confirm the stability of the fcc crystal structure throughout the process, consistently displaying reflections from the {111}, {002}, and {022} planes. However, variations in the intensity and density of the diffraction spots indicate ongoing structural changes, as the crystalline nanoparticles grow, and the surrounding amorphous organic matrix decomposes. Over time, the crystalline nanoparticles show a moderate increase in size from ≈3–5 nm, transitioning into more defined cuboidal morphologies. The morphology development of the particle, along with the thinning of the amorphous matrix, indicates the structural transition driven by sustained electron beam interactions. The gradual decomposition of the organic‐rich amorphous matrix between the nanoparticles eventually leads to a porous structure in certain regions marked by circles in Figure 3e,f. This transition reveals a localized structural evolution from a continuous mixed matrix into a porous structure, consisting of crystalline nanoparticles with residual organic layers surrounding them. The contrast variations observed in the TEM images suggest a 3D (Z‐direction) arrangement of particles along the viewing axis, with some particles appearing out of focus. This observation implies a layer‐by‐layer removal of the amorphous material under sustained electron irradiation, progressively exposing more faceted surfaces of the nanoparticles. Additionally, residual organic layers near the nanoparticles exhibit curved striations pointed by arrows in TEM images, accompanied by an increased intensity in the diffuse halo ring of the amorphous region in FFTs and the emergence of a broad peak in the corresponding radial intensity profile. These curved striations likely result from the stress‐induced short‐range alignment of the remaining polymer‐like chains in the organic material, reflecting the rearrangement of the matrix as it decomposes and interacts with the growing nanoparticles.^[^ ^65^, ^66^, ^67^ ^]^ As crystallization proceeds and more metal atoms incorporate into the lattice, the amorphous matrix becomes less dense and increasingly stretched between particles. These changes are reflected by a slight narrowing of the FFT halo ring and the broad peak of the radial profile up to 40 min. Beyond this timeframe, as the layers degrade and are removed, the curved striations diminish and remain primarily localized around the nanoparticles. Correspondingly, the broad intensity band in FFTs and radial profile plots diminishes.

To further quantify the lattice structure evolution, interplanar distances (d_111_, d_200_, and d_220_) were extracted from the radial profile peaks. The calculated fcc lattice constants (a_111_, a_200_, and a_220_) and their averaged value (a_m_) are plotted in Figure 3h, illustrating lattice parameter variations, while Figure 3i presents the evolution of d‐spacing ratios over time. The overall change in the lattice constant is attributed to the incorporation of metal atoms of varying sizes. An increase in the average lattice size (a_m_) suggests that elements with larger atomic radius progressively integrate into the fcc structure. This observation aligns with the hypothesis that smaller atomic species such as Ni and Co nucleate first, followed by the gradual integration of elements with larger radius, in accordance with their reduction potentials (Ni > Co > Zn > Mn > Mg). Lattice distortion and anisotropic strain are evidenced by the variation among lattice constants in different crystallographic directions and deviations in *d*‐spacing ratios from ideal fcc values (d_111_/d_200_ = 1.155, d_200_/d_220_ = 1.414, and d_111_/d_220_ = 1.633). Specifically, a smaller a_200_ and a larger a_220_ suggest compressive strain along the ⟨100⟩ direction and tensile strain along the ⟨110⟩ direction (Figure 3h). The d‐spacing ratios further support this anisotropy, with the d_111_/d_200_ >1.155, d_111_/d_220_ <1.633, and d_200_/d_220_ <1.414 ratios, directly correlating with the anisotropic strain observed in the lattice constants (Figure 3i). The pronounced deviation in the d_200_/d_220_ ratio, influenced by both reduced d_200_ and increased d_220_ interplanar spacings, highlights significant anisotropic strain between these specific crystallographic directions. This anisotropic strain is primarily driven by the atomic size mismatch inherent to HEAs, where the multi‐element composition induces local lattice distortions.^[^ ^68^ ^]^ Beyond compositional effects, the surrounding amorphous matrix can potentially contribute to the anisotropic strain.^[^ ^69^, ^70^ ^]^ Stress on the polymer‐like chains of the matrix could generate external anisotropic stress fields, enhancing compressive effects on {200} planes and tensile effects on {220} planes. As the matrix decomposes, these external stresses likely diminish, contributing to the observed relaxation of lattice strain.^[^ ^71^ ^]^ The evolution of lattice deviation over time (Figure 3j) provides additional insight into strain dynamics and structural stabilization. During the early stages of crystallization and nanoparticle growth, lattice distortion increases, consistent with atomic size mismatches and the strain introduced by heterogeneous metal incorporation. As the process continues, strain relaxation and structural stabilization occur, likely due to defect diffusion and the diminishing influence of external stresses as the matrix degrades. Notably, the reduction in lattice deviation beyond 45 min aligns with pore formation in the matrix, which can be correlated with a decrease in external stresses as the matrix is removed. It should be noted that compositional (internal) and matrix‐induced (external) strain effects are interdependent and evolve together during the crystallization process. While early‐stage strain reflects the combined impact of atomic size mismatch and external confinement by the amorphous matrix, the subsequent relaxation correlates with matrix decomposition and internal defect dynamics. These effects are not fully separable, but the time‐resolved evolution in Figure 3 supports their dynamic interplay as the origin of the observed anisotropic strain.

STEM‐EDS analysis (Figure S4a, Supporting Information) of the region with crystalline nanoparticles after more than 50 min of electron beam irradiation reveals that the elemental composition closely resembles that of the initial amorphous film (Figure 1d). Elemental mapping confirms co‐localization of Mg, Mn, Co, Ni, and Zn, with a distribution closely matching that of the as‐deposited film and no obvious chemical segregation at the map's resolution. Quantitative analysis of several ≈10 × 10 nm^2^ sub‐regions containing ≈5 nm particles within the probed area shows compositions narrowly clustered around the near‐equimolar target, with compositional differences between regions falling within the range expected from EDS measurement noise and local sampling statistics at this scale (Figure S4b,c, Supporting Information). The element‐wise standard deviations are small, and no element exhibits a systematic enrichment or depletion trend across the measured regions, confirming that, within the detection limits of STEM‐EDS, the crystalline nanoparticles display good overall compositional homogeneity. These results, combined with the structural characterization, indicate that the resulting nanoparticles are quinary MgMnCoNiZn HEAs with a single‐phase fcc structure. The formation of the fcc structure is particularly notable, given the distinct crystalline preferences of the individual elements: Ni and Co naturally form fcc structures, Mn typically crystallizes in a complex body‐centered cubic (bcc) structure, and Mg and Zn favor hexagonal close‐packed (hcp) arrangements.^[^ ^72^ ^]^ Control experiments were conducted using Zn, Mn, and Mg glycerolate films as single metal precursors under similar electron beam conditions. In the Zn and Mn glycerolate films, beam‐induced crystallization yielded hcp Zn nanoparticles (Figure S5a,b, Supporting Information) and α‐Mn nanoparticles with a complex cubic structure (Figure S5c, Supporting Information), rather than the fcc phase observed in the HEA system. In contrast, the Mg glycerolate film showed no detectable metallic Mg even after prolonged beam exposure, consistent with the very low reduction potential of Mg^2^⁺ (E°≈−2.37 V). The film remained largely amorphous, with only sparse and faint crystalline features, and the corresponding diffraction patterns aligned more closely with MgCO_3_ than with metallic Mg (Figure S5d, Supporting Information). The synergistic effect of the elements in the HEA system appears to stabilize the fcc phase, distinguishing it from the single‐element crystallization behavior. Cooperative effects among the constituent elements, particularly those with higher fcc‐forming tendencies (Ni, Co), appear to overcome the structural preferences of the individual metals, directing the formation of a single‐phase fcc alloy that would not emerge from single‐metal precursors alone. As previously mentioned, Ni and Co, being more prone to reduction to zero‐valent metal atoms, likely serve as the initial crystalline nuclei. These nuclei form the foundational crystalline framework and guide the development of the fcc structure in the multielement nanoparticles.

**Figure 4:**
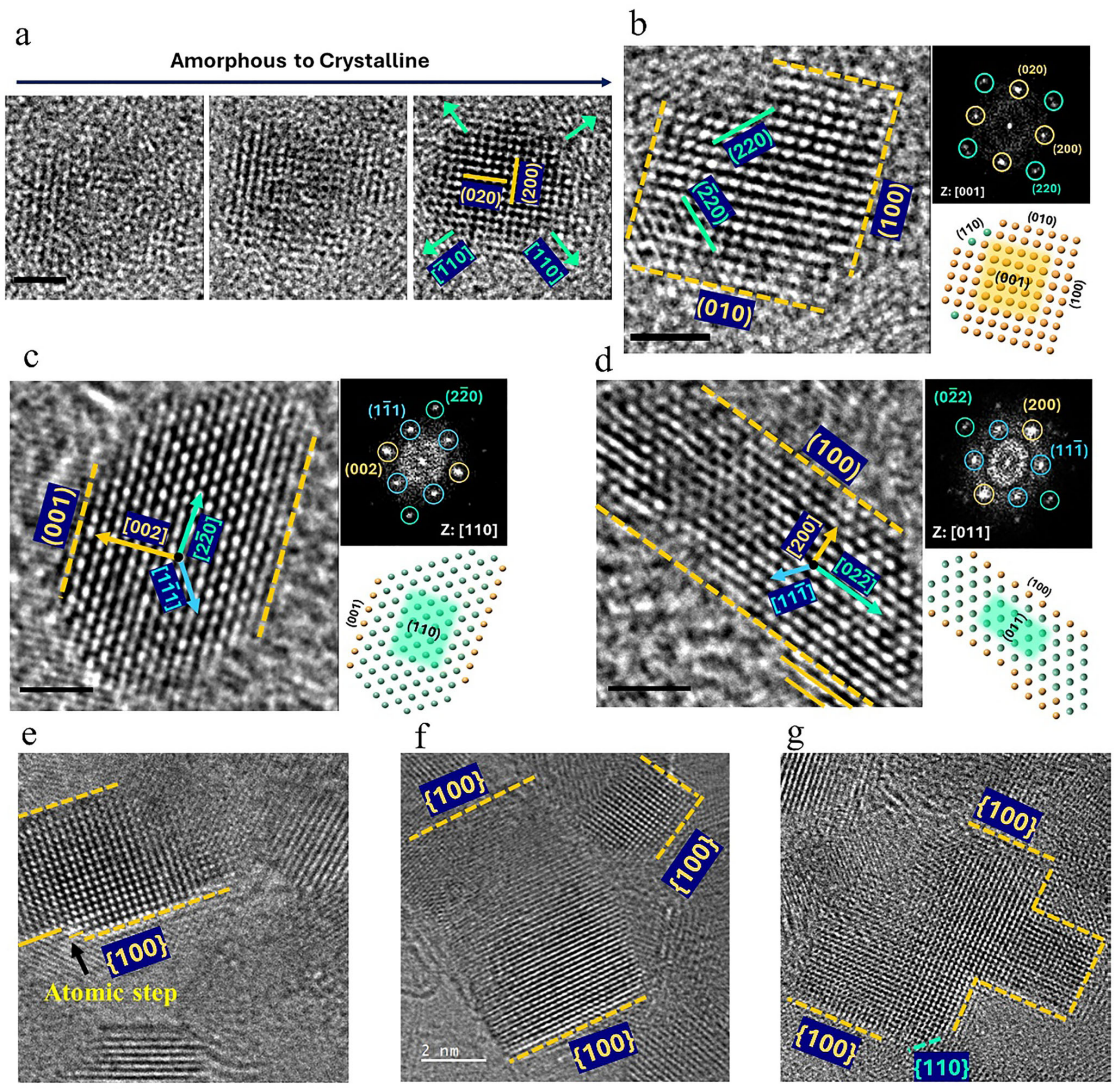
Crystal structure and growth direction of nanoparticles. a) Tracking the HRTEM image of a single particle over time showing the transition from amorphous to crystalline and slow growth in size; b) HRTEM and FFT of the particle growing in (a) showing fcc crystal at [001] direction and facets growing parallel to (200) and (020) planes. c, d) HRTEM image and corresponding FFT of particles at <110> view direction showing facets growing parallel to {100} planes. e–g) HRTEM image of particle at <100> view directions showing the layer growth along the {100} planes.

**Figure** **4** highlights detailed examples of nanoparticles following the crystallization of amorphous HE‐glycerolate film, providing insights into their atomic arrangement, crystallographic orientation, and morphological evolution. The HRTEM images, FFT patterns, and crystallographic models collectively illustrate well‐defined fcc crystal domains with near‐cuboidal morphology oriented along ⟨100⟩ and ⟨110⟩ directions. The sequence of HRTEM images in Figure 4a demonstrates the transformation of a small nanoparticle from an amorphous to a crystalline state. Following crystallization, the nanoparticle grows gradually through surface atomic attachment and rearrangement. The final crystalline state exhibits distinct fcc (200) and (220) lattice planes oriented along the [001] zone axis (Figure 4b). The early‐stage presence of {110} facets suggests that these surfaces initially act as active sites for atomic incorporation, likely due to their higher surface energy and greater density of unsaturated bonding sites.^[^ ^73^ ^]^ However, over time, these facets diminish as the system stabilizes into a morphology dominated by {100} facets.^[^ ^74^ ^]^ For nanoparticles aligned along ⟨110⟩ directions (Figure 4c,d), the images reveal the persistence of stable {100} facets constrained by the amorphous matrix, while the edges parallel to the {220} planes exhibit irregular and less‐defined atomic layers, indicating the atomic attachment in this direction. This observation supports the hypothesis that lattice relaxation occurs through defect migration and atomic rearrangement. The observed lattice distortion along ⟨110⟩ direction further suggests their involvement in strain accommodation, as evidenced by the increased lattice spacing along {220} planes, which gradually decreases during the later stages of growth (Figure 3h). As the amorphous matrix decomposes, compressive strain along ⟨100⟩ direction is relieved, contributing to the stabilization of {100} facets. These findings are consistent with those shown in Figure 3 and with previous studies on fcc metals and HEA nanoparticles, where crystallization typically induces tensile strain along the growth direction and compressive strain perpendicular to it, often leading to slight tetragonal distortions under specific growth conditions or external constraints.^[^ ^75^, ^76^ ^]^

**Figure 5:**
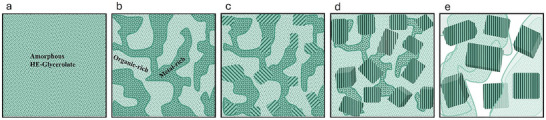
Schematic of the crystallization mechanism of HE‐glycerolate films under electron beam irradiation. a) Initial uniform amorphous film. b) Phase separation into metal‐rich and organic‐rich regions. c) Radiolytic reduction initiates crystalline nucleation. d) Nanoparticles grow as the amorphous matrix decomposes. e) Final cubic nanoparticles surrounded by a porous residual matrix.

Figure 4e‐g presents ⟨100⟩‐oriented nanoparticles at their final growth stages. The presence of atomic‐height surface steps along {100} facets, marked by arrows in Figure 5e, is characteristic of a layer‐by‐layer growth mechanism. In this mode, atomic migration and incorporation occur preferentially at step edges rather than uniformly across the surface, in agreement with classical crystal growth models where layer advancement is governed by the interplay between atomic attachment and surface diffusion kinetics.^[^ ^77^, ^78^, ^79^ ^]^ The formation of cubic nanoparticles with dominant {100} facets can be attributed to a combination of preferential atomic attachment on {110} surfaces, diffusion‐mediated growth on {100} facets, and selective facet stabilization.^[^ ^80^, ^81^ ^]^ In crystalline nanoparticles, direction‐dependent atomic displacement energy influences both defect mobility and strain relaxation.^[^ ^82^, ^83^ ^]^ Prior studies on fcc Ni‐alloys suggest that preferential atomic displacement along {200} planes facilitates the stabilization of extended {100} facets.^[^ ^84^, ^85^ ^]^ Additionally, the final cubic morphology with dominant {100} facets may also be driven by the selective adsorption of polymerized glycerol. Literature reports indicate that polymerized glycerol preferentially adsorbs onto {100} planes due to their lower atomic density and greater availability of coordination sites.^[^ ^86^, ^87^, ^88^ ^]^ The enhanced adsorption of polymerized glycerol from the amorphous matrix on {100} surfaces further stabilizes these facets, promoting the observed cubic morphology. The interplay between atomic displacement, surface reconstruction, and adsorbate‐mediated facet stabilization highlights the controlled nature of electron beam‐induced crystallization, leading to uniform cubic nanoparticles with tailored crystallographic orientations.

To further validate the observed athermal electron beam‐induced crystallization mechanisms, a control experiment was conducted by heating a similar HE‐glycerolate film to 400 °C at a rate of 20 °C min^−1^ using a TEM heating holder. During this process, specific regions of the film were exposed to the electron beam, while others were only heated, without electron beam exposure, to isolate the effects of thermal activation alone. The results, presented in Figures S6 and S7 (Supporting Information), reveal significant differences in crystallization behavior and the size, shape, and composition of final nanoparticles compared to the athermal electron beam‐induced process. In the region subjected to both heating and electron beam irradiation, the HE‐glycerolate film was heated to 400 °C under a lower electron dose rate (10^5^ e nm^−2^ s^−1^) compared to the athermal test to reduce the electron beam effect during the heating time (Figure S6, Supporting Information). Structural transformation proceeded gradually at lower temperatures, with only minor changes up to 200 °C in the first 10 min, but it accelerated significantly after reaching 300 °C at the 15‐min mark. This temperature, which exceeds both the boiling point of glycerol (290 °C) and the activation temperature threshold for thermal diffusion of metal elements,^[^ ^63^ ^]^ triggered rapid structural transformation and nanoparticle growth. By 400 °C and 30 min of heating (Figure S7a,b, Supporting Information), larger (≈20 nm) dark‐contrast particles appeared, dispersed among a denser distribution of smaller (≈5 nm) low‐contrast particles across the film. Elemental mapping of the resulting nanoparticles exhibited pronounced compositional segregation and Ni/Co enrichment in larger particles. Partial Zn depletion (≈6.8 at.% compared to ≈12 at.% in the athermal case) was observed, suggesting volatilization due to its low sublimation energy.^[^ ^89^, ^90^ ^]^ STEM‐EDS mapping revealed that the larger ≈20 nm particles were strongly Ni/Co‐rich (Figures S7d–e, Supporting Information), while Mg, Mn, and Zn were depleted. Quantitative analysis of 15–20 × 20 nm^2^ sub‐regions across the crystallized area (Figures S7f, Supporting Information) showed large compositional variations and systematic enrichment/depletion trends, indicating non‐uniform elemental distribution. In contrast, the region exposed only to heat without electron beam irradiation (Figure S7g,h, Supporting Information) exhibited minimal crystallization, forming only small (≈2 nm) nanoparticles within the amorphous matrix. Elemental mapping (Figure S7i, Supporting Information) shows partial segregation of Co and Ni into crystalline regions, but to a lesser extent than in the combined heat and beam‐induced process. Zn was almost entirely depleted, implying that without the electron beam, it remained weakly bonded in the amorphous matrix and volatilized upon heating. These results contrast sharply with the beam‐induced, no‐heating case, where Zn was retained and no systematic enrichment or depletion was detected (Figure S4, Supporting Information).

These findings highlight the crucial role of the electron beam in accelerating crystallization and influencing elemental retention. The combination of heat and beam exposure facilitated radiolytic reduction of metal ions and enhanced atomic mobility through thermal diffusion, leading to faster crystallization and the aggregation of specific metals (Ni and Co) into larger particles. In contrast, the athermal beam‐induced process, driven by atomic displacement at lower temperatures, restricted atomic mobility to short‐range displacements, thereby maintaining a more uniform elemental distribution and preventing significant phase segregation. The differences in atomic diffusion versus displacement mechanisms explain the contrasting nanoparticle morphologies. Under high‐temperature conditions, long‐range diffusion promotes metal cluster aggregation, reducing control over particle size and shape. Conversely, the athermal process confines growth to localized atomic environments, preventing compositional segregation and enabling a more uniform alloy structure. Furthermore, the slower, displacement‐driven crystallization process supports gradual facet evolution, leading to well‐defined cubic morphologies with exposed {100} facets.

As previously noted, a key challenge in the bottom‐up synthesis of homogeneous HEA nanoparticles from mixed metal salt precursors is overcoming compositional phase segregation caused by variations in elemental reduction activity. In our prior study on liquid‐phase co‐reduction synthesis of colloidal multi‐element alloy nanoparticles, we demonstrated that more reactive elements nucleate first, triggering sequential reduction and crystal growth. This effect is exacerbated in liquid‐phase synthesis due to high atomic mobility. To counteract this issue, many approaches focus on limiting reaction space and restricting elemental mobility. Using a pre‐mixed amorphous matrix in a sol‐gel‐based method is a promising strategy to hold multi‐element ions together and suppress de‐mixing. However, for this technique to be effective in HEA synthesis, crystallization conditions must be carefully controlled. Metallic nanoparticle crystallization from this precursor requires a reducing environment and cannot be achieved solely through thermal calcination, which is commonly used in ceramic synthesis. The thermally assisted electron beam‐induced method faces a challenge similar to that of liquid‐phase synthesis: at high temperatures, enhanced atomic mobility promotes the aggregation of primary metal atoms into clusters, while the delayed reduction of less reactive elements leads to secondary particle formation and phase segregation. Additionally, the high rate of atomic attachment compared to surface atomic diffusion results in uncontrolled nanoparticle shapes and morphologies. On the other hand, the low‐temperature electron beam‐induced crystallization of an amorphous precursor demonstrates the benefits of spatially restricted multi‐element mobility. This short‐range diffusion process minimizes major phase and compositional separation, enabling the formation of single‐phase metallic nanoparticles composed of diverse metal elements. These findings underscore the importance of diffusion‐limited environments in achieving homogeneity in HEA nanoparticle synthesis.

The crystallization mechanism of HE‐glycerolate films under electron beam irradiation follows a sequential transformation, as illustrated schematically in **Figure** **5**. The process begins with a homogeneous amorphous film (Figure 5a) composed of polymerized metal ion glycerolate nanosheets, maintaining a uniform distribution of metal ions and organic components with no distinct structural ordering. This amorphous phase serves as the foundation for subsequent transformations under electron beam exposure. Upon irradiation, phase separation occurs (Figure 5b), leading to the formation of distinct metal‐rich and organic‐rich regions. This phase segregation creates localized environments conducive to crystallization, as metal ions cluster together while the organic matrix undergoes structural rearrangement. The emergence of these domains marks the onset of structural evolution. Crystallization initiates within the metal‐rich regions (Figure 5c), where radiolytic reduction of metal ions leads to the formation of small metallic nuclei. These initial seeds expand into well‐defined crystalline nanoparticles under continued irradiation. As the process advances (Figure 5d), the nanoparticles increase in size and develop more defined morphologies, accompanied by the thinning and degradation of the surrounding amorphous matrix. The transition from irregular clusters to distinct crystallites signifies the dominance of atomic rearrangement in nanoparticle formation. As the crystallization process progresses further, the residual amorphous matrix continues to decompose (Figure 5e), facilitating the continued growth and stabilization of the crystalline nanoparticles. The resulting nanoparticles predominantly adopt cubic morphologies with exposed {100} facets, a consequence of surface energy minimization and selective facet stabilization. The progressive decomposition of the organic matrix contributes to the development of a porous structure surrounding the nanoparticles, marking the completion of the crystallization process. This amorphous‐to‐crystalline transition, resulting in the formation of HEA nanoparticles dispersed within the matrix, creates a dual‐phase structure and composite material. The porosity that develops within the structure during the phase transformation further adds to the functional versatility of the material, making it suitable for a wide range of applications. In particular, the intimate interface between metal domains and the residual carbonaceous matrix provides high surface area, interconnected porosity, and efficient charge‐transfer pathways, features that are highly advantageous for catalysis and energy applications.^[^ ^91^, ^92^, ^93^ ^]^ Such amorphous/crystalline heterogeneity has been shown to enhance accessibility to active sites, facilitate reactant diffusion, and suppress nanoparticle aggregation, thereby improving phase stability and long‐term durability under electrochemical conditions.^[^ ^93^, ^94^, ^95^, ^96^ ^]^ Similar porous hybrid architectures have demonstrated promising performance in electrocatalysis and water splitting, including thermally decomposed metal–organic films, bifunctional high‐entropy systems, and amorphous heterophase nanosheets, suggesting that the structures formed here may hold comparable potential, even though direct functional testing lies beyond the scope of this study.

## 3 Conclusion

This study reveals a two‐step crystallization mechanism for HEA nanoparticles formed from amorphous HE‐glycerolate films under electron beam irradiation. The process initiates with beam‐induced phase separation, followed by radiolytic metal ion reduction and localized atomic rearrangement into a crystalline structure. Real‐time TEM observations show that this transformation leads to the formation of uniform fcc nanoparticles with cuboidal morphology and dominant {100} facets. The direct visualization of this sequential transformation highlights the unique capabilities of athermal electron beam processing to decouple nucleation and growth, offering fine control over crystallization pathways in multicomponent systems. These findings not only clarify the mechanisms behind beam‐induced alloy crystallization but also highlight a broader principle: limiting atomic mobility during multielement crystallization is essential for suppressing segregation and achieving uniform alloy phases. As such, this approach provides a valuable foundation for designing new synthesis routes for multicomponent materials where controlled phase and shape are critical. While in situ TEM synthesis is presently limited to microscale volumes, the mechanistic insights gained in this study, especially the role of confined atomic mobility and sequential radiolytic reduction, can inform the development of scalable low‐temperature processing strategies for homogeneous HEA materials. Moreover, the formation of HEA nanoparticles within an amorphous matrix creates a dual‐phase, porous composite with enhanced structural and functional versatility as a potential approach for designing HEA‐based materials for applications in catalysis, energy storage, and structural systems.

## 4 Experimental Section

### Chemicals

Glycerol, IPA, and nickel acetate tetrahydrate (Ni (CH_3_COO)_2_·4H_2_O) were purchased from Sigma‐Aldrich. Cobalt acetate tetrahydrate (Co (CH_3_COO)_2_·4H_2_O), magnesium acetate tetrahydrate (Mg (CH_3_COO)_2_·4H_2_O), and zinc acetate dihydrate (Zn (CH_3_COO)_2_·2H_2_O) were purchased from Synth. Manganese acetate tetrahydrate (Mn (CH_3_COO)_2_·4H_2_O) was purchased from Carlo Erba. All the chemicals were used as received without any additional purification.

### HE‐Glycerolate Synthesis

Equimolar amount of Ni (CH_3_COO)_2_·4H_2_O, Co (CH_3_COO)_2_·4H_2_O, Mn (CH_3_COO)_2_·4H_2_O, Mg (CH_3_COO)_2_·4H_2_O and Zn (CH_3_COO)_2_·2H_2_O (0.5 mmol each) were dissolved in 40 mL IPA under continuous stirring for 10 min for the alcoholysis (metal ion dispersion and binding to the OH groups of the solvent). Then, 8 mL of glycerol (C_3_H_8_O_3_) was added to the mixture as a metal‐organic complexation agent, resulting in a viscous solution containing metal ions coordinated with polymerized glycerol after stirring for 2 h. The mixture solution was then transferred to a Teflon‐lined stainless‐steel autoclave and kept at 180 °C for 1 h to undergo a solvothermal process forming HE‐glycerolates.

### In‐situ TEM experiment

For the in‐situ TEM study, the condensation and gel formation are achieved by dropping the solution on a lacy carbon Copper TEM grid. The liquid solution on the grid was dried at ambient conditions, and a gel layer was deposited on the grid as the initial sample for the real‐time observation of a room‐temperature formation of a nanocrystalline structure under an electron beam. In situ TEM observation was carried out on a JEOL ARM200CF Microscope operating at 200 kV and 5 × 10^6^ e nm^−2^ s^−1^ electron dose rate. Videos were acquired using an Orios SC200D CCD and ClearView Gatan camera with 10 frames per second at a magnification of ×2 M. For the in situ heating experiment, the solution was drop‐cast onto Protochips Fusion thermal chips, membrane‐supported holey carbon films. Afterward, the dedicated Fusion Select heating holder was loaded into the microscope to run heating experiments with temperature accuracy over 95% for TEM analysis. The precursor film was heated to 400 °C at a ramp rate of 20 °C min^−1^ with a selected area subjected to electron beam irradiation at a dose rate (10^5^ e nm^−2^ s^−1^).

### EDS Characterization of Composition

The elemental composition of the as‐deposited gel layer and a locally beam‐induced transformed area with a nanocrystalline grain was characterized at the end of the process. Energy dispersive spectroscopy (EDS) was performed using an aberration‐corrected JEOL ARM200CF with a cold field emission gun operated at 200 kV, equipped with an Oxford X‐max 100TLE windowless X‐ray detector.

### Image Analysis

FFT‐derived d‐spacings were obtained from calibrated HRTEM images by radial intensity profile analysis using ImageJ software. The FFT sampling interval sets a precision of ≈0.01–0.02 Å, and repeated measurements across different regions gave reproducibility within ±0.01–0.02 Å (≈0.3%–0.5%). Lattice parameters were calculated from these d‐spacings, and uncertainties were propagated to lattice constants and their ratios, giving an overall error of ≈0.5%.

## Conflict of Interest

The authors declare no conflict of interest.

## Supporting information



Supporting Information

## Data Availability

The data that support the findings of this study are available in the supplementary material of this article.
