# Supplementary material for: In Situ Visualization of Electron Beam‐Driven High‐Entropy Alloy Crystallization
Source: Adv Sci (Weinh). 2025 Oct 21;13(2):e12587. doi: 10.1002/advs.202512587 (PMC12786330; doi:10.1002/advs.202512587)
Supplement: Supplementary file 1 — Supporting Information [file ADVS-13-e12587-s001.docx]

**Supplementary Information**

**In Situ Visualization of Electron Beam-Driven High-Entropy Alloy Crystallization**

Azadeh Amiri^1^, Reza Shahbazian-Yassar^1*^

^1^Department of Mechanical and Industrial Engineering, University of Illinois Chicago, Chicago, 60607, IL, USA.

E-mail: rsyassar@uic.edu


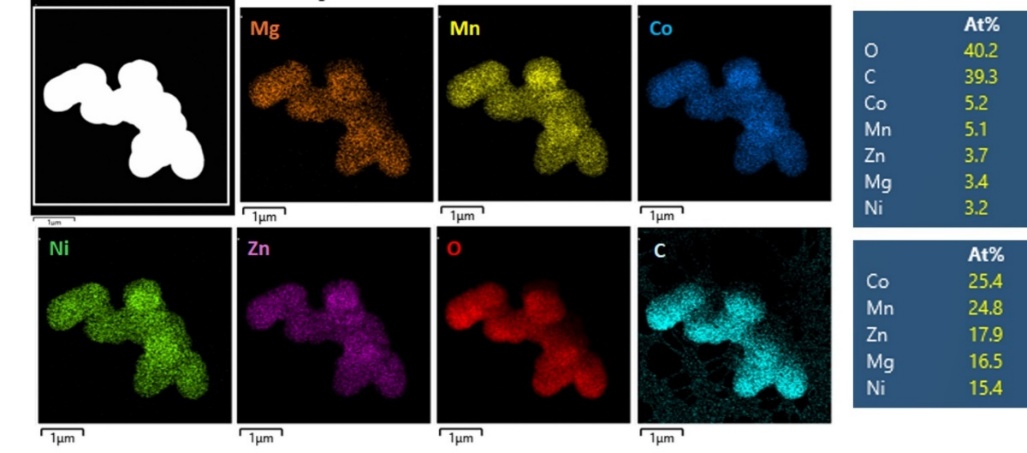


**Figure S1**. STEM-EDS elemental mapping distribution and atomic composition of spherical amorphous HE-glycerolate particles obtained after 6 hr. solvothermal process at 180 ^◦^C.


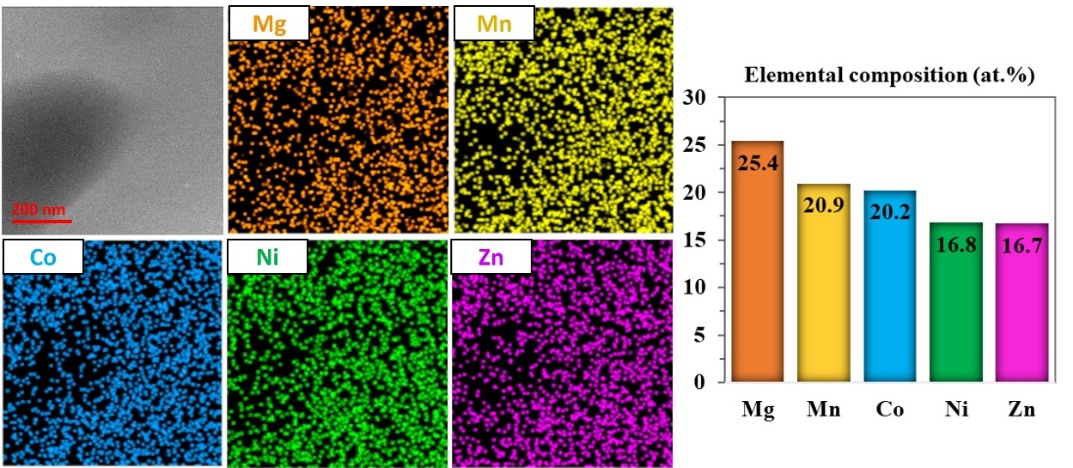


**Figure S2**. STEM-EDS elemental map of distribution of elements and quantitative analysis of elemental composition of deposited film of HE-Glycerolate solution after 1 year storage at closed vial at ambient condition.

**Table S1. Properties of elements**  ^1–7^

**
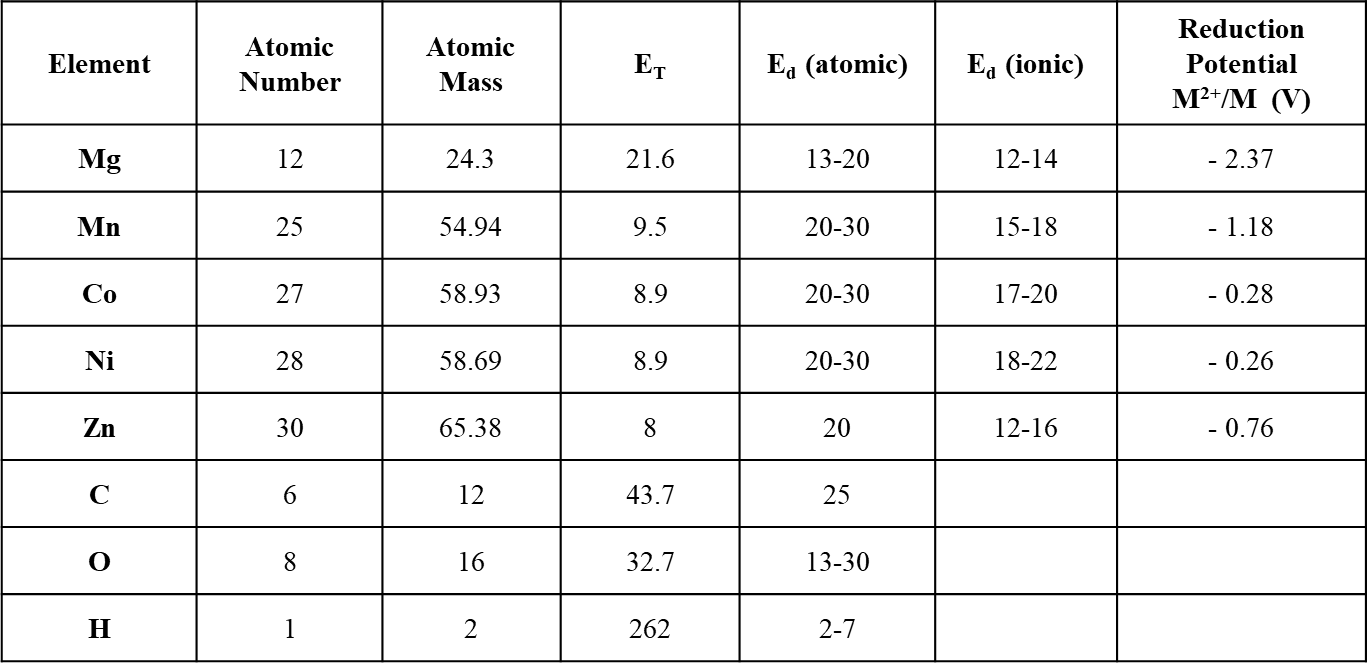
**


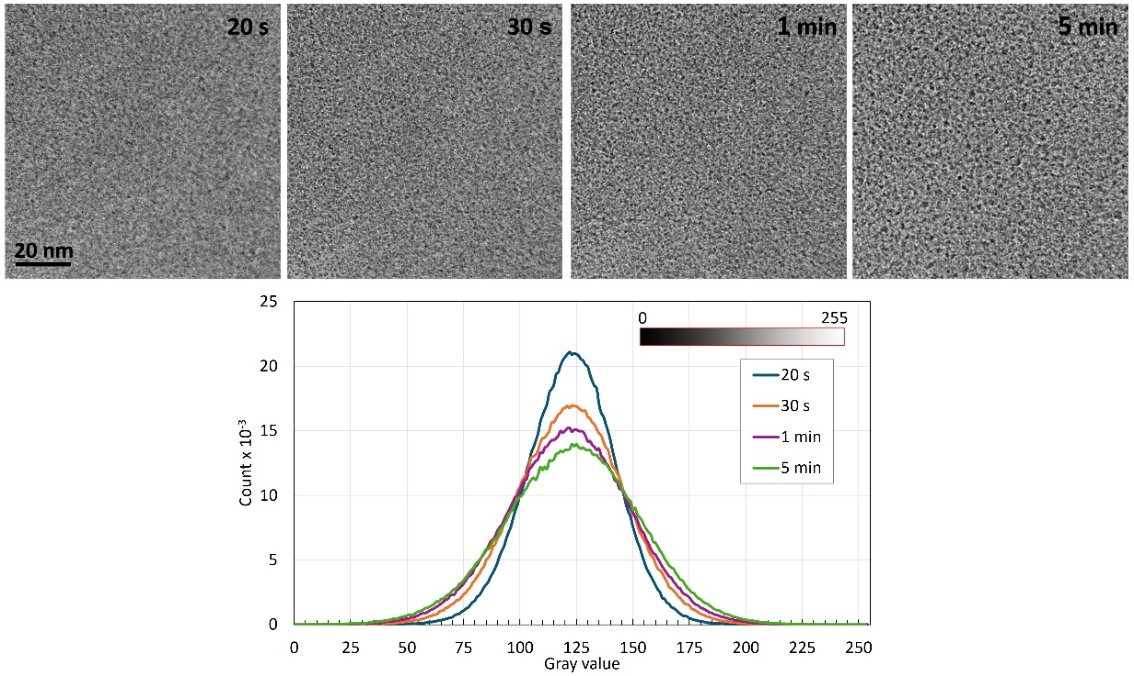


**Figure S3.** TEM images of HE-glycerolate films exposed to electron beam irradiation for different durations (20 s, 30 s, 1 min, and 5 min), showing progressive structural evolution. Below, the corresponding grayscale histograms illustrate the distribution of pixel intensities for each exposure time. The shift in the histogram peak and distribution indicates changes in contrast and density, suggesting structural modifications over time.


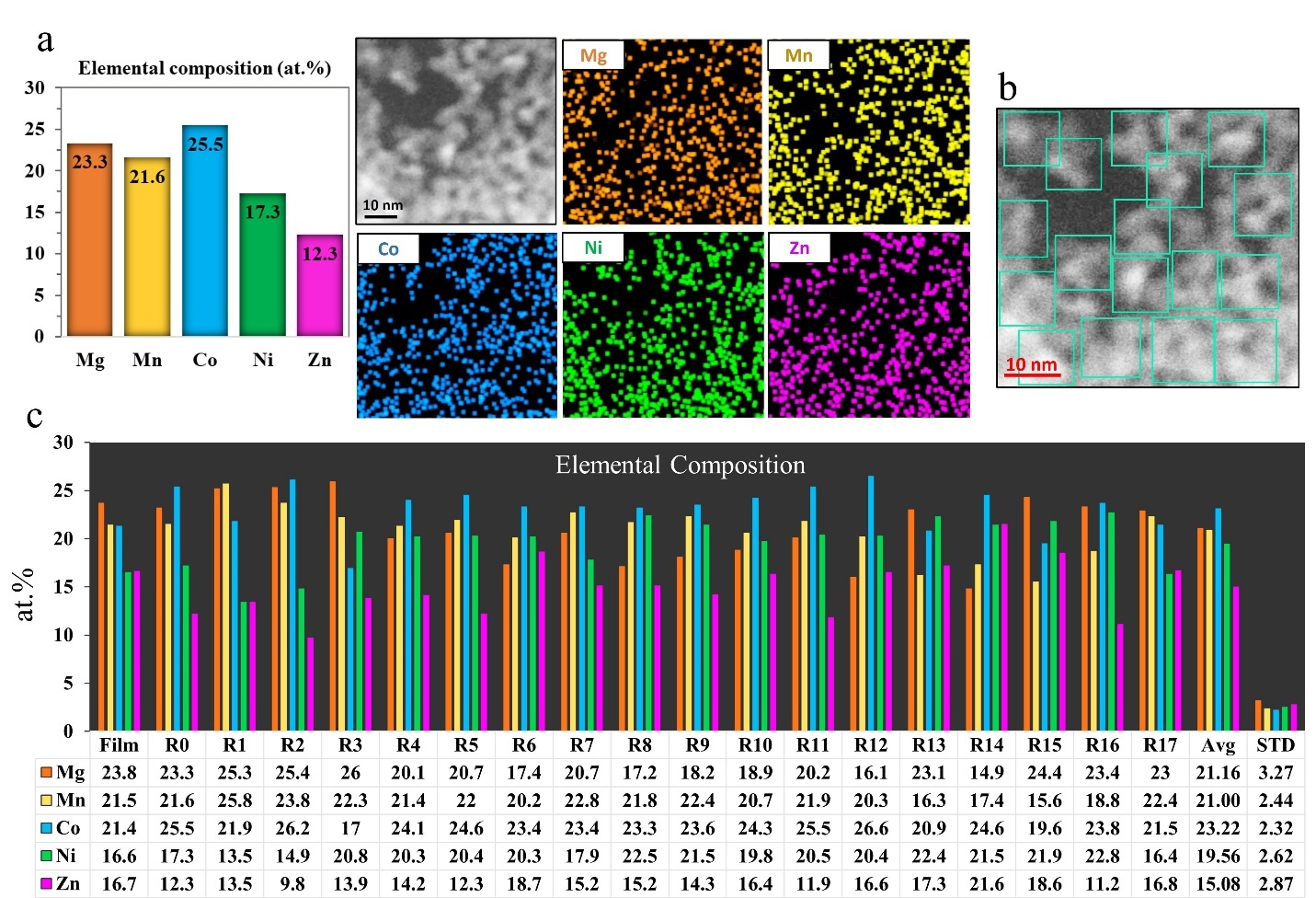


**Figure S4**. STEM-EDS analysis of multielement HEA nanoparticles. (a) Quantitative atomic composition and elemental maps of the crystalline region after >50 min of electron beam irradiation. (b) STEM image of the probed area (~60 × 60 nm², R0) with 17 smaller subregions (~10 × 10 nm², R1–R17) outlined in green for additional quantitative spectrum analysis. (c) Elemental composition results from EDS quantification of the initial amorphous film, the full crystalline region, and each subregion, along with the calculated average and standard deviation.


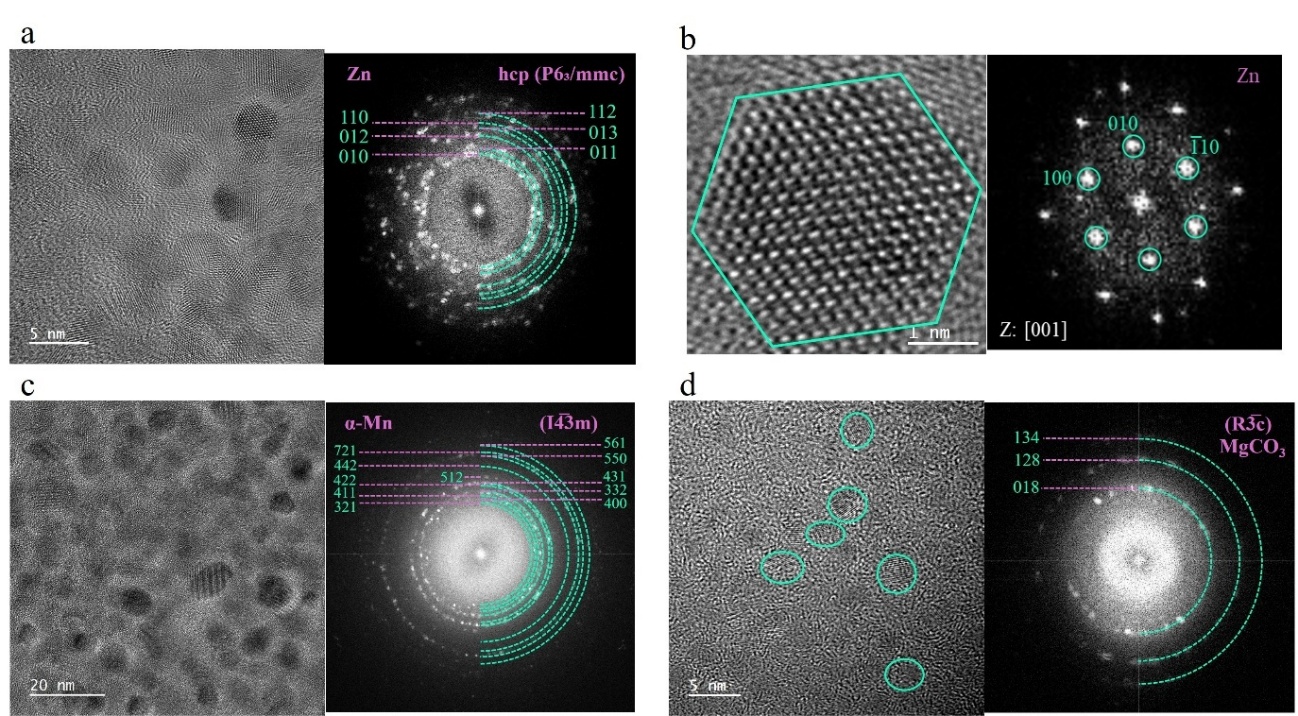


**Figure S5.** Electron beam-induced crystallization of single-element glycerolate film precursors. (a) TEM image and corresponding FFT pattern of Zn-glycerolate film after irradiation, showing nanoparticles with hcp Zn structure (P6₃/mmc). (b) High-resolution TEM and FFT of a single Zn nanoparticle oriented along the [001] zone axis, confirming its hexagonal symmetry. (c) TEM and FFT of Mn-glycerolate film revealing α-Mn nanoparticles with complex cubic structure (I4̅3m). (d) TEM image and FFT of Mg-glycerolate film after irradiation, showing a mainly amorphous matrix with faint crystalline fringes consistent with MgCO₃ (R3c).


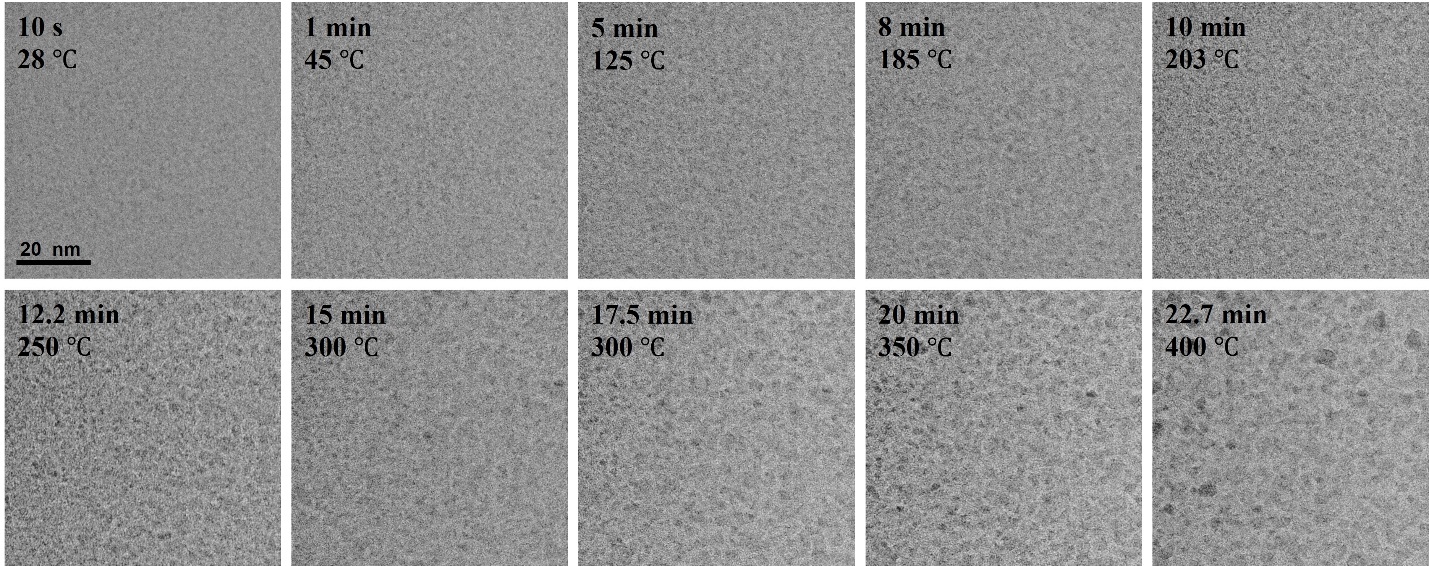


**Figure S6.** Time-sequence TEM images depicting the crystallization process of the HE-glycerolate film under combined electron beam irradiation and in situ heating, recorded at increasing exposure durations and corresponding temperatures. Progressive structural evolution and nanoparticle formation are observed as both exposure time and temperature increase, culminating in fully developed nanoparticles by ~400 °C.


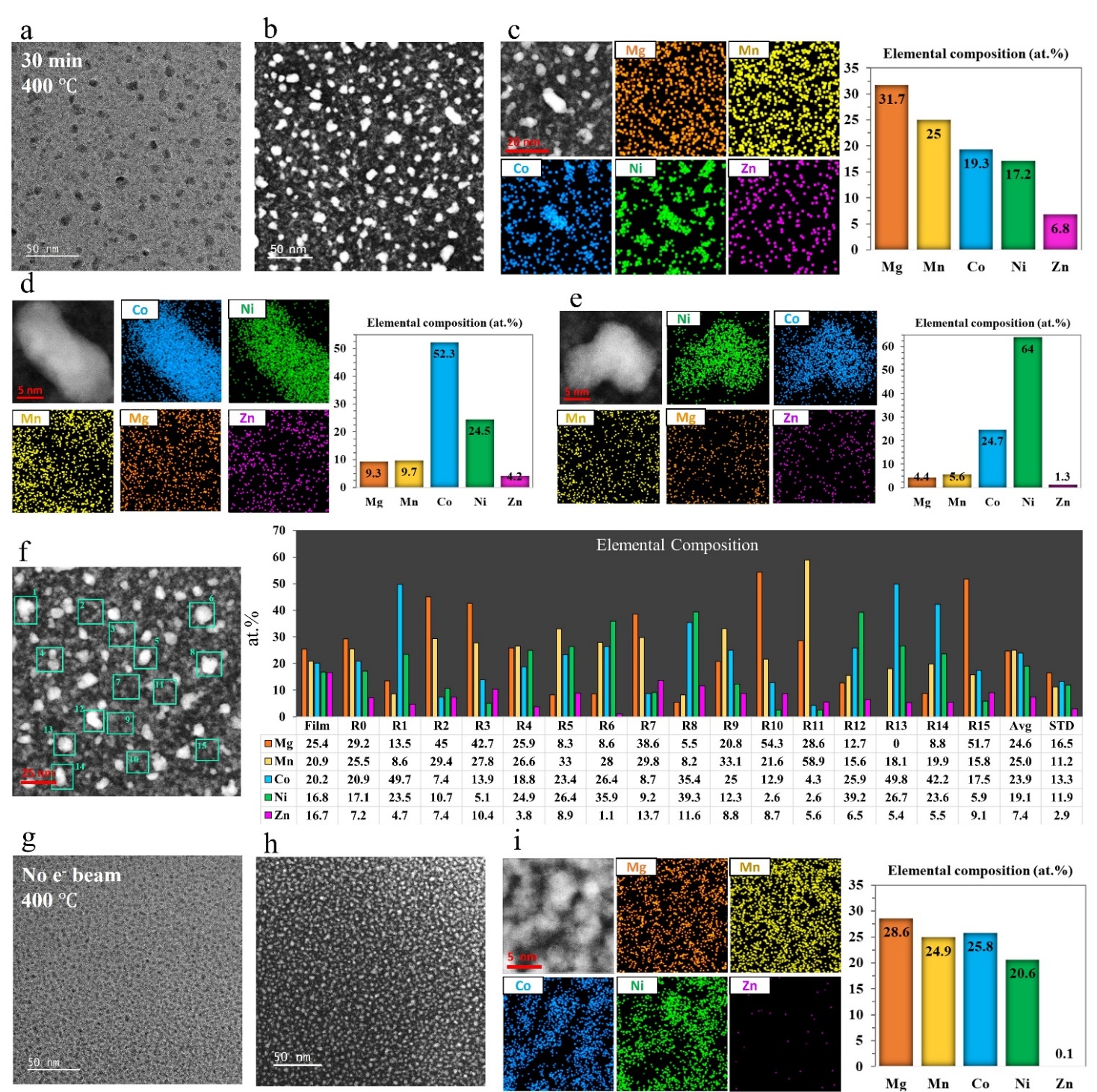


**Figure S7.** (a) TEM and (b) STEM images of the film after 30 min of electron beam irradiation at 400°C, showing distinct nanoparticle formation. (c) EDS elemental mapping and atomic composition analysis of the crystallized nanoparticles after electron beam irradiation, revealing the distribution of Mg, Mn, Co, Ni, and Zn. (d,e) EDS elemental mapping and atomic composition analysis of individual large (~20 nm) Ni/Co rich particles. (f) STEM image of the probed area (~150 × 150 nm², R0) with 15 smaller subregions (~20 × 20 nm², R1–R15) outlined in green and their corresponding quantitative EDS Elemental composition analysis results of each subregion the full crystalline region(R0) and the initial amorphous film (Figure S2), and, along with the calculated average and standard deviation.(g) TEM and (h) STEM images of the film heated to 400°C without electron beam exposure, showing a significantly different particle size. (i) EDS elemental mapping and atomic composition analysis of the sample heated without electron beam irradiation, highlighting differences in elemental distribution compared to the irradiated sample.

**References**

1. Egerton, R. F., Li, P. & Malac, M. Radiation damage in the TEM and SEM. *Micron* **35**, 399–409 (2004).

2. Konobeyev, A. Y., Fischer, U., Korovin, Y. A. & Simakov, S. P. Evaluation of effective threshold displacement energies and other data required for the calculation of advanced atomic displacement cross-sections. *Nucl. Energy Technol.* **3**, 169–175 (2017).

3. Cathala, G., Brunel, C., Chappelet Tordo, D. & Lazdunski, M. Bovine kidney alkaline phosphatase. Catalytic properties, subunit interactions in the catalytic process, and mechanism of Mg2+ stimulation. *J. Biol. Chem.* **250**, 6046–6053 (1975).

4. Homocianu, M., Airinei, A., Ipate, A. M. & Hamciuc, C. Spectroscopic Recognition of Metal Ions and Non-Linear Optical (NLO) Properties of Some Fluorinated Poly(1,3,4-Oxadiazole-Ether)s. *Chemosensors* **10**, 183 (2022).

5. Ray, W. J. & Mildvan, A. S. Role of Bivalent Cations in the Phosphoglucomutase System. IV. a Study of the mn2+ Binding Site by Means of Nuclear Relaxation Measurements on Water Protons. *Biochemistry* **9**, 3886–3894 (1970).

6. Shen, Y. F., Suib, S. L. & ƠYoung, C. L. Effects of Inorganic Cation Templates on Octahedral Molecular Sieves of Manganese Oxide. *J. Am. Chem. Soc.* **116**, 11020–11029 (1994).

7. Garau, A. *et al.* [9]aneN3-based fluorescent receptors for metal ion sensing, featuring urea and amide functional groups. *Dalt. Trans.* **48**, 4949–4960 (2019).
